# Patellar Tendon Fibroma Mimicking Hemangioma: A Report on a Rare Case

**DOI:** 10.7759/cureus.66607

**Published:** 2024-08-10

**Authors:** Khalid Ibrahim, Osama Elhag, Amna AlKtebi, Ahmed AbouHelwo, Rabia Farhan

**Affiliations:** 1 Radiology, Hatta Hospital/Dubai Academic Health Corporation, Dubai, ARE; 2 Radiology, Medical Fitness/Dubai Academic Health Corporation, Dubai, ARE; 3 Orthopaedic Surgery, Hatta Hospital/Dubai Academic Health Corporation, Dubai, ARE; 4 Pathology, Dubai Hospital/Dubai Academic Health Corporation, Dubai, ARE

**Keywords:** soft tissue tumours, spindle-shaped cells, tumors around knee joint, tendon sheath fibroma, patellar tendon

## Abstract

Fibroma of the tendon sheath (FTS) is an uncommon benign soft tissue tumor of the tendon sheath. Clinical and radiological features are not distinctive enough to clinch the diagnosis preoperatively. FTS occurs mostly around small joints such as the fingers, hands, and wrist. However, it rarely arises around a large joint (knee, shoulder, elbow, or ankle). This case report describes a rare presentation of fibroma within the patellar tendon. The patient, a 35-year-old male, presented with progressive pain and swelling in his left knee. Clinical examination, imaging studies, and histopathological analysis mimicked hemangioma but confirmed the diagnosis of a patellar tendon fibroma. A surgical excision was performed, leading to significant improvement in symptoms and functional recovery. This case highlights the importance of considering rare soft tissue pathologies in the differential diagnosis of knee joint disorders.

## Introduction

Fibroma of the tendon sheath (FTS) is defined as a slow-growing nodular neoplasm adjacent to the tendon sheath, clinically manifested by a small joint mass or effusion that predominantly affects people between the third and sixth decade of age, affecting mainly men, and more commonly the small joints of the fingers and hand, being relatively rare in large joints [1,2]. The knee is the most common location of the large joints, mostly associated with the cruciate ligament and posterior capsule, with only a few cases described in the literature of tendon sheath tumors arising from the infrapatellar sheath [3]. The purpose of this report is to further illustrate this rare clinical entity through the introduction of an additional case and to provide readers with a complete literature review. It is expected that this paper could serve as a useful guide for future clinicians and investigators in knowing how this knee joint tendon sheath fibroma would present itself clinically and how to achieve an accurate diagnosis in a proper manner.

## Case presentation

A 35-year-old male presented to the orthopedic clinic with a history of progressively increasing pain in his left knee over the last few months (on and off). The patient reported no significant trauma and denied any previous surgery. Physical examination revealed a palpable mass along the lateral aspect of the knee joint, with associated tenderness of the patellar tendon. However, the knee joint showed a full range of movement.

**Figure 1:**
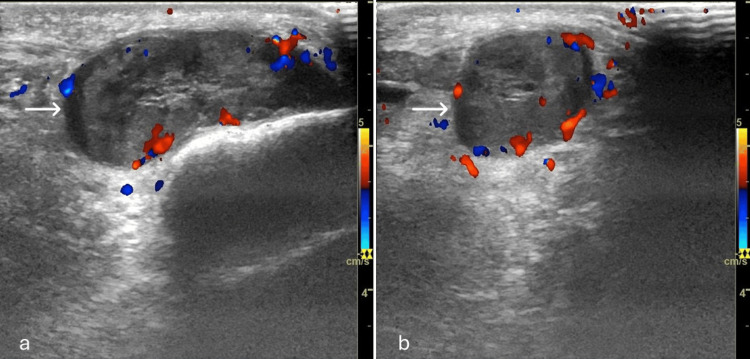
Ultrasound images of the left knee joint: (a) longitudinal and (b) transverse Showing a well-defined oval-shaped solid soft tissue lesion (arrows) in the antero-lateral infrapatellar region measuring (4 cm × 1.6 cm). The lesion is of mixed echogenicity and showed internal vascularity. There are no calcifications/cystic changes.

**Figure 2:**
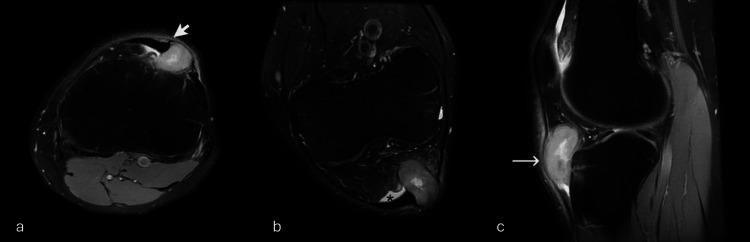
MRI images PD FS (a) axial, (b) coronal oblique, and (c) sagittal Showing soft tissue mass (long arrow) splaying the lateral part of the patellar tendon with claw sign (short arrow) with some edema in the adjacent infrapatellar fat tissue (asterisk).

**Figure 3:**
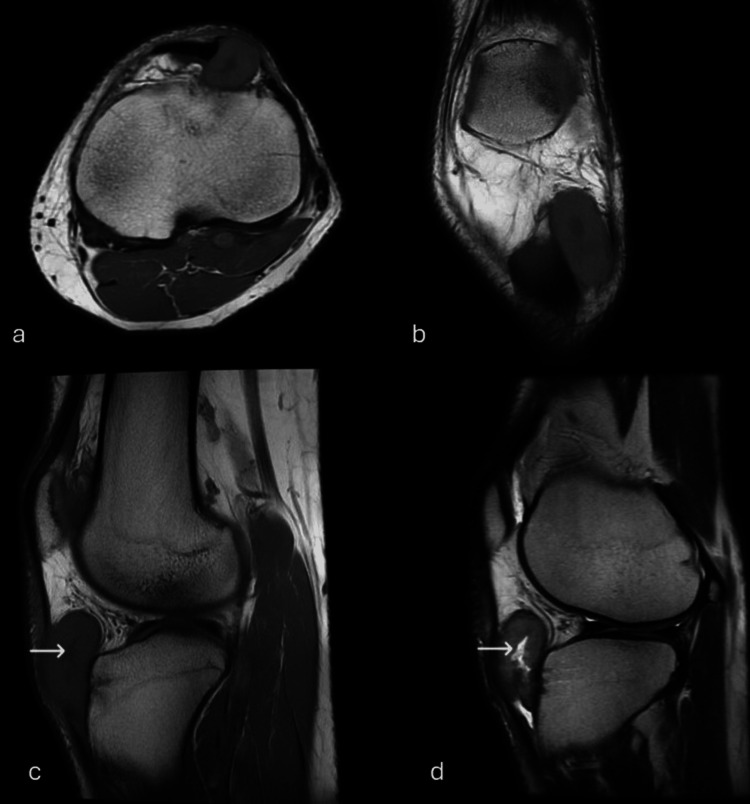
(a) Axial, (b) coronal, (c) sagittal T1 images, and (d) sagittal T2 image Showing the mass with a slightly high signal on T1W and heterogeneously high signal on T2W image, there is central area showing high T2 and low T1 signal (white arrows).

**Figure 4:**
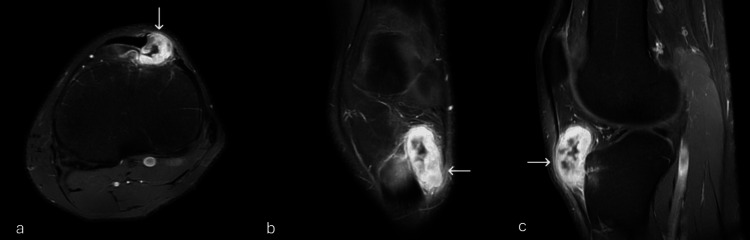
(a) Axial, (b) coronal, and (c) sagittal fat-suppressed T1 post contrast images Showing the avid enhancement of the mass (white arrows). The central area is not enhancing mostly fluid content.

Imaging studies, including ultrasound and magnetic resonance imaging (MRI), demonstrated a well-defined infrapatellar mass showing rather high signal on T1W and heterogeneously high signal on T2W images compared to the muscles with a central linear area of high signal in T2W and low signal on T1W. The mass showed avid heterogeneous contrast enhancement and exerted a minimal mass effect on Hoffa’s fat pad with minimal edema. The mass is seen splaying the lateral part of the patellar tendon, denoting tendon sheath origin with no intraarticular involvement (Figures 1-4).

Surgical intervention

Given the progressive symptoms and imaging findings, the decision was made to surgically excise the mass. Intraoperatively, a firm, encapsulated mass (4 cm × 1.5 cm) was identified lateral to the patellar tendon. A complete excision of the mass was achieved without compromising the ligament integrity. In the postoperative course, the patient was complaining of mild left knee pain and swelling that regressed gradually as the knee joint showed a full range of movement, so the patient was discharged 72 hours after the surgery with a plan of follow-up at the orthopedic clinic.

Post-surgical follow up

**Figure 5:**
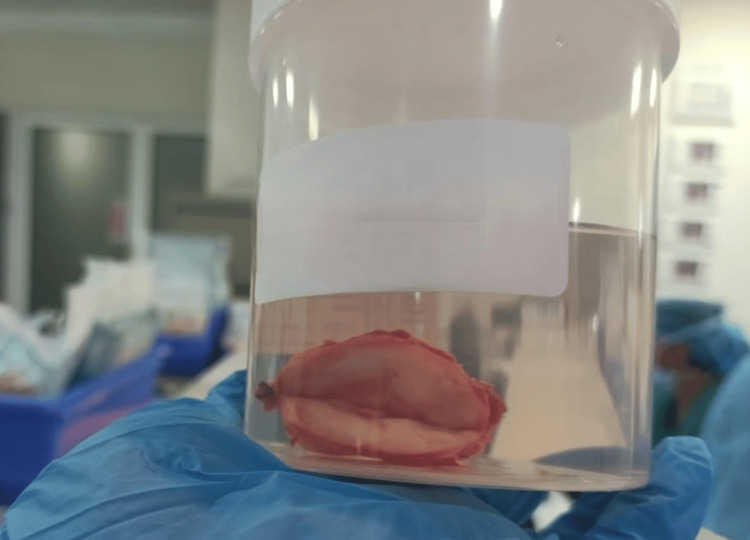
Post-surgical specimen Showing the excised patellar tendon encapsulated mass (4 cm × 1.5 cm).

The patient had three visits to the orthopedic clinic after the surgery. The first one was a follow-up visit, while the last two were for different complaints (right elbow pain). The left knee was pain-free during all visits, with no swelling or palpable mass (Figure 5).

Histopathological analysis

**Figure 6:**
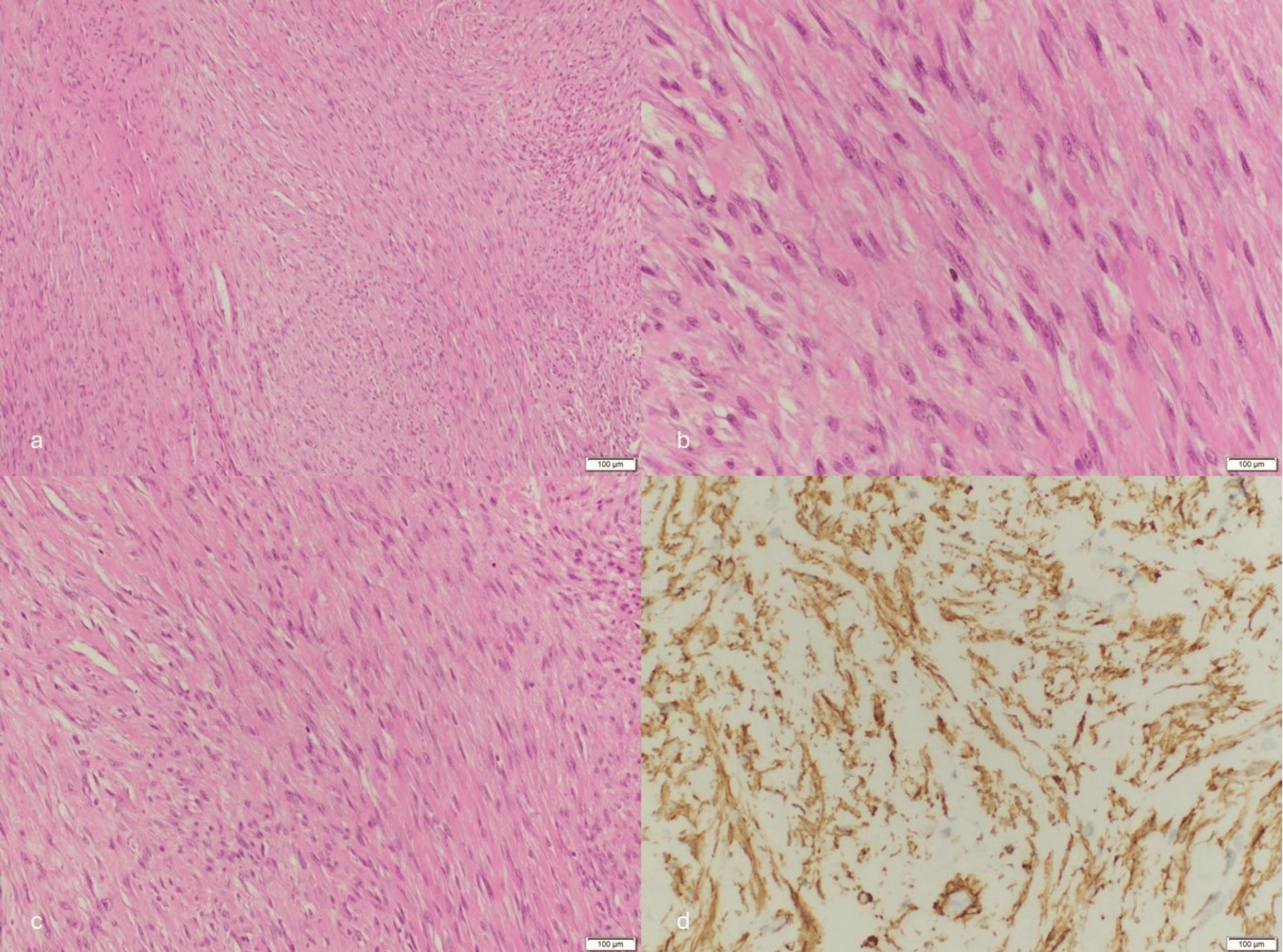
Histological examination of fibroma of the tendon sheath (a) Low-power view shows proliferation of spindle cells; (b) and (c) high-power views show spindle cells with uniform nuclei; (d) SMA immunostain positive staining in spindle cells.

Histopathological examination revealed a well-circumscribed neoplasm composed of an intermixed proliferation of spindle- or stellate-shaped cells with interspersed thin-caliber vascular channels against a collagenous or fibrous background. The cells are bland, with elongated nuclei showing no pleomorphism or hyperchromasia. No giant cells are seen. No areas of necrosis or mitosis were seen. The differential diagnosis includes fibroma of the tendon sheath and hemangioma. An immunohistochemistry profile was done, and the diagnosis of fibroma of the tendon sheath was confirmed (Figure 6).

## Discussion

Geschickter and Copeland described tendon sheath fibroma (FTS) in 1949 [4], and Chung and Enzinger published the largest series of 138 cases, reporting their clinical and pathological features [5]. FTS can be defined as a benign fibroblastic nodular neoplasm originating from the tendon sheath synovium [6].

Macroscopically, the lesion appears well-defined with lobulation. Histopathologically, it contains spindle- or stellate-shaped cells that look like fibroblasts within a thick layer of collagenous stroma. In most cases, cytological atypia is absent. While the majority of lesions are hypocellular, on occasion, certain lesions exhibit increased cellularity in the peripheral region, which is similar to nodular fasciitis [7]. The presence of elongated, thin-walled vessels or clefts, also known as slit-like spaces, is a specific histological finding associated with FTS [7]. FTS affects patients in their second to fifth decades of life and is more common in men than in women, with a ratio of three to one [5]. It is described as presenting as a small, slowly growing mass that is painless [5-7]. The tendons and tendon sheaths of the small joints like fingers (47.9%), hands (24.8%), and wrists (10.3%) are mostly affected [8]. Rarely is FTS reported around large joints (2.8-4.2%), such as the elbows, shoulders, hips, knees, and ankles [8,9].

The initial diagnostic procedure for tendon sheath fibroma in the knee joint involves a clinical examination to rule out any local neurological deficit, bony pathology, or swelling. Ultrasonography and magnetic resonance imaging are helpful for differential diagnosis. A final diagnosis can only be made after a histopathological examination. If the tumor is sufficiently removed, surgery is the preferred course of treatment, and local recurrence is rare.

Imaging

Generally, CT scans and radiographs are normal. Tumor characteristics and location can be diagnosed with an MRI. Typically, FTSs on T1-weighted images exhibit iso- to low signal intensity [10]. On T2-weighted images, FTSs exhibit a range of signal alterations. Low-signal-intensity tumors on T2-weighted images typically exhibit significant fibrous elements and marked hypocellularity [11]. Consequently, the majority of FTSs have low signal intensities on T2-weighted images [9,12-14]. However, in some FTSs, the hypointense areas are mixed with high signal intensity [1,15-17]. Fox et al. stated that the histological characteristics of FTS were reflected in the MRI findings [10]. Occasionally, the tumor displayed a centrally high signal intensity and a peripherally slightly low signal on T2-weighted imaging. According to these findings, the tumor had myxoid changes and central regions of increased cellularity set against a background of slit-like vascular [10]. Different kinds of gadolinium-enhanced MRI findings are observed in different cases. The enhancement patterns can be rim/peripheral [16,18-20], patchy or focal [2,9,21], or lobular enhancement. Takakubo et al. [16] reported peripheral enhancement, which could reflect blood vessel growth at the periphery of the tumor. Pinar et al. stated that the presence of synovium surrounding an intra-articular FTS is reflected by rim enhancement [18].

## Conclusions

Tendon sheath fibromas are rare soft tissue tumors that can occur in various anatomical locations. Although uncommon, these tumors should be considered in the differential diagnosis of knee joint pathologies, especially when associated with a palpable mass and imaging findings consistent with a soft tissue tumor.

This case report underscores the importance of considering rare soft tissue pathologies in the evaluation of knee joint disorders. A thorough clinical examination, appropriate imaging studies, and histopathological analysis are crucial for accurate diagnosis and subsequent management. Surgical excision and post-surgical follow-up, as demonstrated in this case, can lead to favorable outcomes and the resolution of symptoms.
